# Discovery and serological validation of DAMP-derived B-cell epitopes as diagnostic biomarkers for diabetic nephropathy

**DOI:** 10.3389/fendo.2025.1652922

**Published:** 2025-11-24

**Authors:** Chengyuan Yu, Yishi Dong, Fuhua Zhong, Jun Zeng, Zhiye Fang

**Affiliations:** 1 Guangdong Provincial Clinical Research Center for Geriatrics, Shenzhen Clinical Research Center for Geriatrics, Department of Geriatrics, Shenzhen People’s Hospital (The First Affiliated Hospital, Southern University of Science and Technology, The Second Clinical Medical College, Jinan University), Shenzhen, China; 2 Clinical Research Center, Shenzhen People’s Hospital (The First Affiliated Hospital, Southern University of Science and Technology, The Second Clinical Medical College, Jinan University), Shenzhen, China; 3 Center for Medical Experiments, Shenzhen Guangming District People’s Hospital, Shenzhen, China; 4 Division of Respiratory Medicine, Shenzhen Guangming District People’s Hospital, Shenzhen, China

**Keywords:** HMGB1, S100A8, S100A9, B-cell epitopes, diabetic nephropathy, peptide-based ELISA, serological biomarkers, peptide-based diagnostics

## Abstract

**Background:**

Diabetic nephropathy (DN), a severe complication of diabetes and a leading cause of end-stage renal disease, is strongly associated with chronic inflammation triggered by damage-associated molecular patterns (DAMPs), such as high-mobility group box 1 (HMGB1), S100A8, and S100A9. This study aimed to identify and validate conserved B-cell epitopes from these DAMPs to develop peptide-based serological markers for DN diagnosis.

**Methods:**

Canonical sequences of HMGB1 (NP_002119.2), S100A8 (NP_002955.1), and S100A9 (NP_002956.2) were retrieved from NCBI RefSeq. Evolutionary conservation was assessed using MAFFT v7.520 and ConSurf. Linear epitopes were predicted with BepiPred 2.0 (threshold = 0.5) and ABCpred (threshold = 0.51), while conformational epitopes were mapped using ElliPro (score ≥ 0.5, distance ≤ 6Å) on crystallographic structures (PDB: 2YRQ, 1XK4). Candidate epitopes were evaluated for antigenicity (VaxiJen v2.0, threshold = 0.5), surface accessibility (DSSP > 20%), and cross-reactivity (BLASTp against UniProtKB/Swiss-Prot 2024_03). Top peptides were synthesized via Fmoc-SPPS (≥ 95% purity, confirmed by HPLC/MS) and validated using indirect enzyme-linked immunosorbent assay (ELISA) on sera from DN patients (*n* = 30) and healthy controls (*n* = 30). Diagnostic performance was assessed via receiver operating characteristic (ROC) analysis.

**Results:**

Three highly immunogenic and evolutionarily conserved linear B-cell epitopes were identified: HMGB1 (GSSGMGKGDPKKPRGK, VaxiJen = 1.60), S100A8 (NSIIDVYHKYSLIKGN, 1.20), and S100A9 (SVKLGHPDTLNQGEFK, 0.70). These epitopes overlapped with predicted conformational regions and were confirmed to be surface-exposed through structural modeling. ELISA analysis revealed significantly elevated IgG responses in DN patient sera versus controls (*p* < 0.01), with the HMGB1-derived peptide showing the most robust and specific immunoreactivity, highlighting its strong diagnostic potential.

**Conclusion:**

This study successfully identified and validated three novel DAMP-derived B-cell epitopes with significant diagnostic potential for diabetic nephropathy. The peptides exhibited high immunogenicity, strong specificity, and consistent performance in ELISA-based serological assays. These findings pave the way for the development of noninvasive, peptide-based diagnostic tools for early DN detection. Future efforts will focus on multicenter validation and integration into multiplex serological panels.

## Introduction

Diabetic nephropathy is one of the serious and common microvascular complications of both diabetes type 1/2, affecting approximately 30%–40% of diabetic patients and remaining a primary cause of end-stage renal disease (ESRD) globally (1, 2). It is clinically defined by persistent albuminuria, reduced glomerular filtration rate (GFR), and histopathological features such as glomerular basement membrane thickening, mesangial matrix expansion, and tubulointerstitial fibrosis (3, 4). Despite decades of research, current diagnostic tools, such as urine albumin-to-creatinine ratio (UACR) and estimated GFR (eGFR), detect the disease only after substantial renal injury has already occurred. These biomarkers lack sensitivity for early detection and are influenced by several nonrenal factors, such as hypertension, urinary tract infections, and dehydration, rendering them suboptimal for timely diagnosis and prognosis (5). Recent studies indicate that sterile inflammation plays a central role in DN pathogenesis, beyond the traditional metabolic and hemodynamic mechanisms (6). Damage-associated molecular patterns (DAMPs)—endogenous molecules released from stressed or necrotic cells—have emerged as key drivers of sterile inflammation in the diabetic kidney (7). DAMPs activate pattern recognition receptors (PRRs), including Toll-like receptors (TLRs) and the receptor for advanced glycation end products (RAGE), thereby initiating innate immune responses and propagating inflammatory cascades (8). Among these, high-mobility group box 1 (HMGB1), S100A8, and S100A9 have gained attention, as they are markedly upregulated in diabetic renal tissues and also detected at elevated levels in patient serum (9, 10). These DAMPs not only contribute to renal damage but may also serve as immunogenic molecules that elicit adaptive immune responses, including the production of autoantibodies (7). They represent promising targets for diagnostic biomarker development, particularly in the form of B-cell epitopes. B-cell epitopes are discrete regions on antigens recognized by antibodies and can be linear (continuous) or conformational (discontinuous) (11). Identifying conserved, surface-exposed, and immunogenic B-cell epitopes within HMGB1, S100A8, and S100A9 would allow the design of synthetic peptides capable of detecting DN-specific antibodies in patient sera (12). Compared to full-length proteins, peptides offer advantages in terms of production cost, stability, and reduced nonspecific binding, making them ideal candidates for use in enzyme-linked immunosorbent assay (ELISA)-based diagnostic platforms. The integration of computational prediction tools with structural bioinformatics has proven invaluable for epitope discovery. Tools such as BepiPred 2.0 and ABCpred enable accurate prediction of linear B-cell epitopes based on machine learning and physicochemical parameters, while ElliPro supports conformational epitope prediction using 3D protein structures. Moreover, combining antigenicity assessment (e.g., VaxiJen), surface accessibility (Emini scale), and conservancy analysis across isoforms ensures that selected epitopes are both biologically relevant and broadly applicable in diverse patient populations (13). However, only a limited number of studies have attempted to combine these computational tools with experimental validation for DN-specific biomarker development. A recent systematic review emphasized the lack of noninvasive, early-stage biomarkers for DN that directly reflect molecular damage (14). Furthermore, studies have shown that patients with DN develop circulating antibodies against renal DAMPs, but the specificity and diagnostic utility of such responses remain largely unexplored (15). In light of these gaps, epitope-based peptide biomarkers could offer a transformative diagnostic strategy, enabling the detection of subclinical disease before the onset of irreversible kidney damage. Therefore, the present study aimed to identify conserved, surface-exposed B-cell epitopes derived from HMGB1, S100A8, and S100A9 using a robust immunoinformatics pipeline, followed by validation of their structural and antigenic relevance. The shortlisted peptides were subsequently assessed for their serological performance in distinguishing DN patient sera from healthy controls using ELISA. By integrating *in silico* epitope prediction with experimental validation, this study provides a foundation for the development of peptide-based, noninvasive diagnostic tools for the early detection of DN.

## Materials and methods

### Ethical approval and sample collection

All human serum samples were obtained in accordance with the institutional ethical committee (IEC) of Shenzhen People’s Hospital guidelines and approved. Written informed consent was obtained from all participants prior to sample collection. The study was conducted in full accordance with the ethical principles of the Declaration of Helsinki. We collected serum samples from 30 biopsy-confirmed diabetic nephropathy patients (mean age 58 years ± 7 years; eGFR < 60 mL/min/1.73 m²; UACR > 300 mg/g) and 30 age-matched healthy controls. Following consent, venous blood was drawn into clot-activated tubes, processed within 2 h (2,000×*g*, 15 min, 4°C), and stored at −80°C until analysis.

### Sequence retrieval and conservation analysis

Reference protein sequences for HMGB1 (NP_002119.2), S100A8 (NP_002955.1), and S100A9 (NP_002956.2) were retrieved in FASTA format from the NCBI RefSeq (accessed 11 May 2024) Protein Database (https://blast.ncbi.nlm.nih.gov/Blast.cgi?PAGE=Protein), selecting only canonical human isoforms to ensure consistency. Accession numbers were recorded for reproducibility. To assess evolutionary conservation and reduce inter-individual variability, multiple sequence alignment (MSA) was performed using MAFFT version 7.520 with the G-INS-i algorithm and BLOSUM62 scoring matrix. Evolutionary conservation scores were calculated using ConSurf (scale 1–9), with residues scoring ≥ 7 considered highly conserved and prioritized for subsequent B-cell epitope prediction.

### B-cell epitope prediction and structural modeling

Both linear and conformational B-cell epitopes were predicted using a multitool approach. Linear epitopes were identified using BepiPred 2.0 (random forest classifier, threshold = 0.5) and ABCpred (recurrent neural network, threshold = 0.51), which apply machine learning algorithms trained on known antigen–antibody complexes. Conformational epitopes were predicted using ElliPro via The Immune Epitope Database (IEDB) server with default settings (minimum score = 0.5; maximum distance = 0.6 Å), employing 3D protein structures obtained from the Protein Data Bank (HMGB1: 2YRQ; S100A8/A9: 1XK4) (https://www.rcsb.org/). Structural validation included solvent accessibility analysis (DSSP relative accessibility > 20%), antigenicity prediction (VaxiJen v2.0, human-specific model, threshold 0.5), and electrostatic potential mapping (APBS with 150 mM ionic strength).

### Homology screening and peptide synthesis

Potential epitopes were screened against UniProtKB/Swiss-Prot 2024_03 using BLASTp (*E*-value < 0.01, > 30% identity over ≥ 8 residues, exclusion criteria applied). Three top epitopes (HMGB1_150–165_, S100A8_42–57_, S100A9_88–103_) were synthesized via Fmoc-based solid-phase peptide synthesis (CEM Liberty Blue™) and purified by RP-high-performance liquid chromatography (HPLC; Waters, Milford, Massachusetts XBridge C18, 5%–60% ACN gradient). Peptide identity and purity (> 95%) were confirmed by LC-MS (Agilent, Santa Clara, California 6545 Q-TOF, ESI+).

### Peptide synthesis and ELISA validation

Three top-ranking B-cell epitopes (one per protein: HMGB1, S100A8, S100A9) were chemically synthesized via Fmoc-based solid-phase peptide synthesis (SPPS), achieving ≥ 95% purity as confirmed by HPLC and mass spectrometry. For indirect ELISA, 96-well plates were coated with each peptide at an optimized concentration of 10 µg/mL in carbonate-bicarbonate buffer (pH 9.6) and incubated overnight at 4°C. The optimal coating concentration was determined through preliminary titration experiments ranging from 1 to 20 µg/mL, selecting the concentration with the highest signal-to-noise ratio and minimal nonspecific binding. Plates were blocked with 5% BSA in PBS-T (0.05% Tween-20) for 1 h at room temperature. Serum from DN patients (*n* = 30) and healthy controls (*n* = 30) was applied at a 1:100 dilution and incubated for 2 h at room temperature. HRP-conjugated anti-human IgG (1:5,000) was used for detection, followed by Tetramethylbenzidine (TMB) substrate, and absorbance was read at 450 nm using a BioTek ELx800™ microplate reader. To ensure assay specificity, a scrambled peptide of similar length and amino acid composition was included as a negative control. Each sample was assayed in triplicate. Statistical significance was determined using unpaired *t*-tests, with *p* < 0.05 considered significant.

## Results

### Protein sequence retrieval and conservation analysis

The canonical protein sequences of HMGB1 (NP_002119.2; 215 aa), S100A8 (NP_002955.1; 93 aa), and S100A9 (NP_002956.2; 114 aa) were retrieved from NCBI RefSeq (accessed 05-11-2024). MAFFT v7.520 alignment (G-INS-i algorithm, BLOSUM62 matrix) of human isoforms revealed evolutionary conservation patterns, with ConSurf analysis identifying 12 functionally critical regions (scores ≥ 7; **Supplementary Figure S1**). Notably, the HMGB1 DNA-binding domain (residues 150–165) exhibited the highest conservation (score = 8.9), while S100A8/A9 calcium-binding loops (S100A8_42–57_, S100A9_88–103_) showed scores ≥ 7.5, suggesting structural and functional constraints.

### Linear and conformational epitope prediction

To enhance the precision and robustness of B-cell epitope identification, an integrated computational approach was employed. Predictions were combined from two complementary tools: BepiPred 2.0 (Area Under Curver (AUC) = 0.85) and ABCpred (AUC = 0.67). BepiPred 2.0, which utilizes a random forest algorithm trained on known antibody–antigen structural data, was applied with a default threshold of 0.5 to identify high-probability linear epitope regions (**Supplementary Tables S1–S3**). In parallel, ABCpred, based on artificial neural networks designed to predict 16-mer linear epitopes, was applied with a threshold of 0.51 to ensure algorithmic diversity in epitope prediction (**Supplementary Table S4**). To ensure diagnostic relevance and biological consistency, the predicted epitopes were mapped onto conserved regions identified through multiple sequence alignment. Only epitopes aligning with highly conserved segments across human isoforms were prioritized for downstream analysis. This conservation check was crucial to ensure that the selected peptides are not only immunogenic but also stable across genetic variants, increasing their diagnostic reliability in a diverse population and minimizing the risk of false negatives due to sequence variability. Ultimately, peptides predicted by both tools and located within conserved regions were shortlisted for synthesis and experimental validation. This rigorous selection pipeline strengthened confidence in the diagnostic potential of the identified peptide candidates by integrating antigenicity, sequence conservation, and prediction consensus.

**Figure 1:**
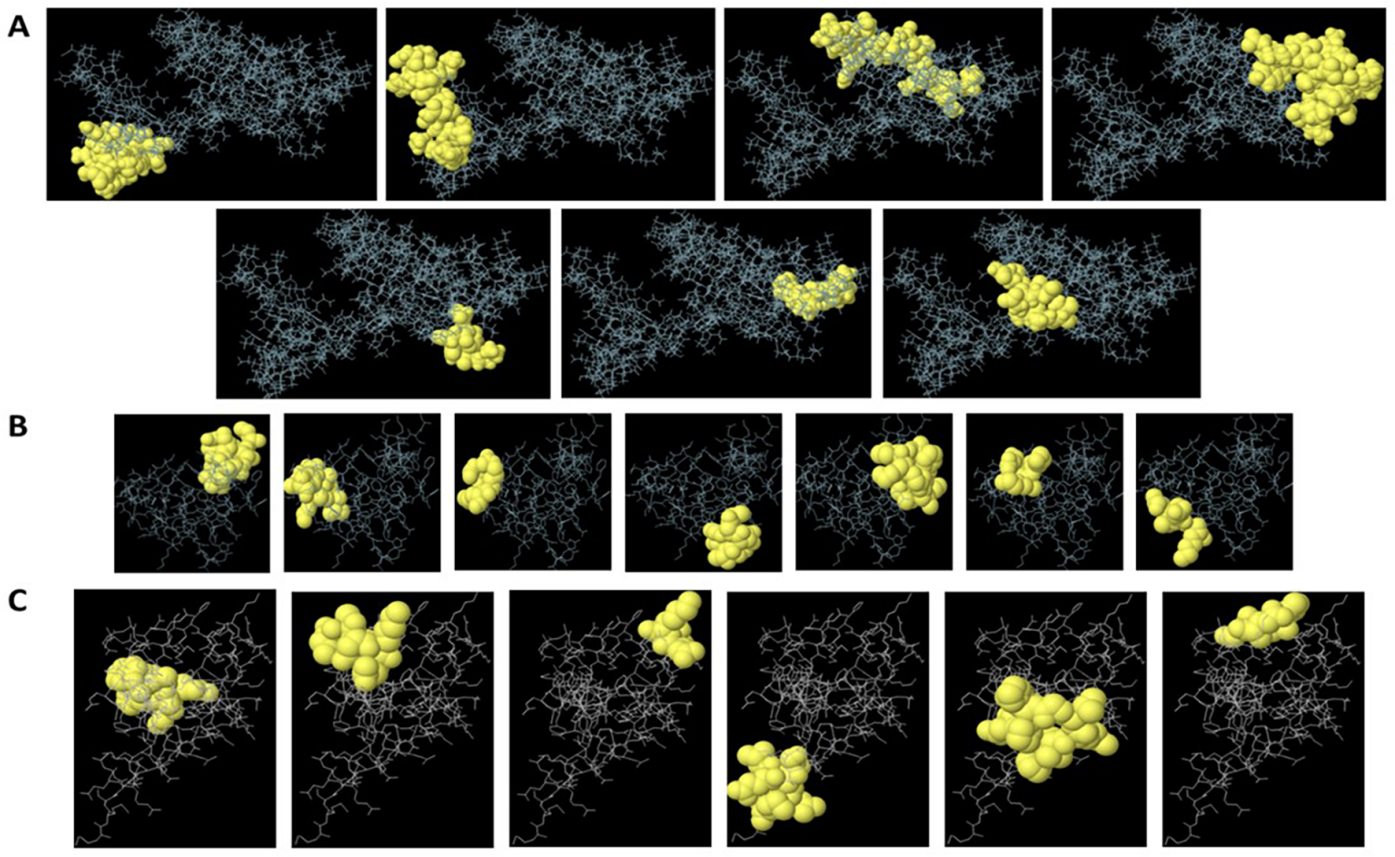
Predicted linear and conformational B-cell epitopes mapped onto HMGB1 **(A)**, S100A8 **(B)**, and S100A9 **(C)** structures. Epitopes predicted by BepiPred 2.0, ABCpred, and ElliPro are visualized on the 3D structures of HMGB1 (PDB: 2YRQ) and S100A8/S100A9 (PDB: 1XK4). Overlapping linear and discontinuous epitope regions are highlighted, with key residues labeled to illustrate spatial clustering.

ElliPro analysis of the target protein structures (Protein Data Bank [PDB]: 2YRQ, 1XK4) predicted four high-confidence discontinuous epitopes (score ≥ 0.7). The top-scoring epitope (score 0.82) localized to HMGB1, comprising residues 150–165 and 189–195 (**Figure 1**, **Supplementary Figure S2**). Spatial clustering of these residues (mean Cα distance: 4.2 Å ± 0.8 Å) confirmed their structural proximity, indicating a conformationally stable epitope. Three additional epitopes (scores ≥ 0.7) were identified, albeit with lower scores than the HMGB1 epitope (**Supplementary Figure S2**). ElliPro’s predictions highlighted surface-exposed, antibody-accessible regions not identifiable through linear epitope analysis alone. Strong overlap with previously identified linear regions was observed for the predicted discontinuous epitopes, reinforcing their selection for validation. The overlap of conformational and linear predictions further increased confidence in the surface accessibility and immunogenic potential of the selected peptides.

### Structural validation and antigenicity

**Figure 2:**
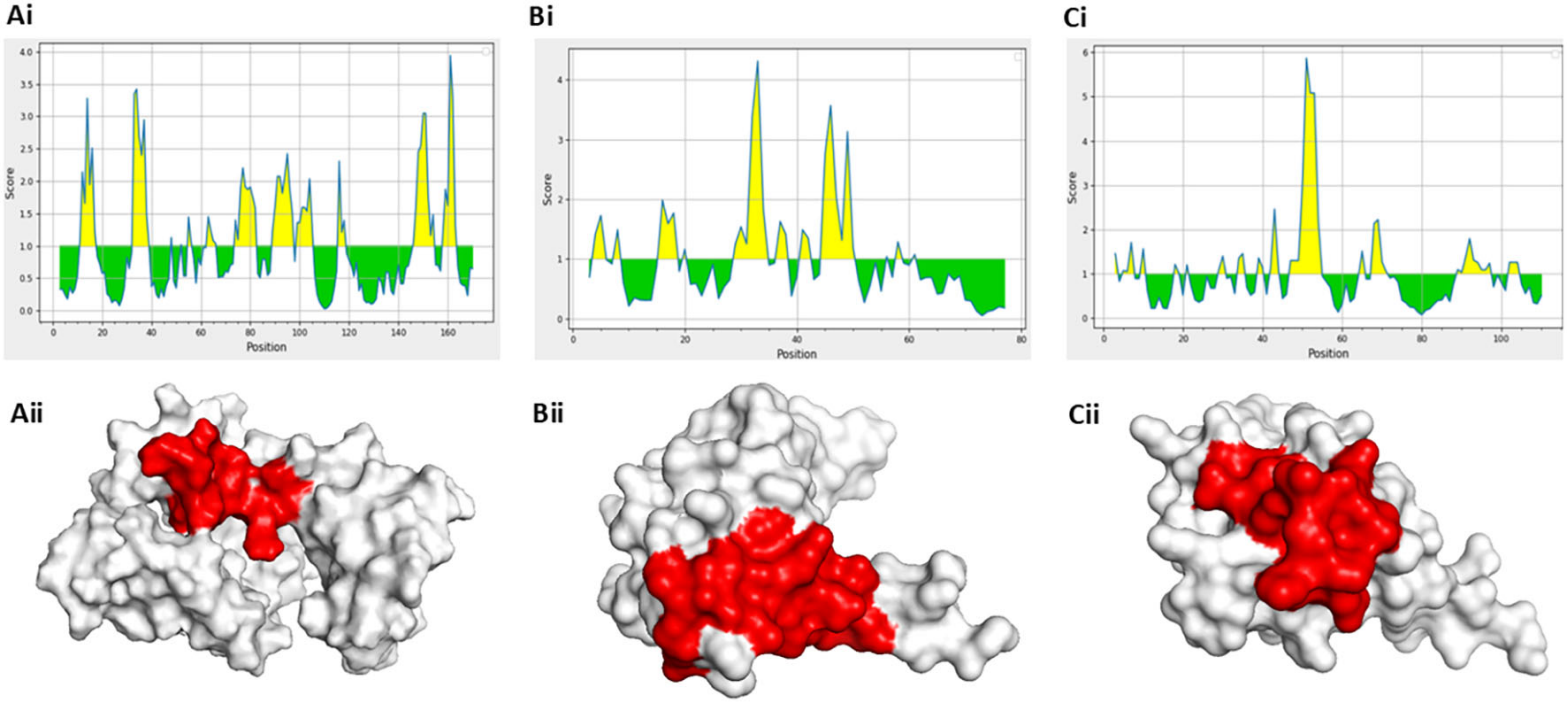
Surface accessibility and structural localization of selected peptides using Emini and PyMOL analyses. **(a [i]–c [i])** Surface accessibility profiles of selected epitopes predicted by Emini surface accessibility prediction. **(a [ii]–c [ii])** 3D structural visualization of peptide localization in solvent-exposed regions using PyMOL.

First, the surface accessibility of the predicted epitopes was evaluated using the Emini Surface Accessibility Prediction tool available on the IEDB server to validate their extracellular exposure. This tool calculates the probability of each amino acid residue being accessible on the protein surface, a crucial feature for antibody recognition. The shortlisted epitopes were located in surface-exposed regions, as confirmed by the resulting profiles, indicating their potential serologically detectable markers (**Figures 2A–C**). In parallel, 3D structural models of the target proteins were obtained from the PDB: 1XK4 for S100A8 and S100A9, and 2YRQ for HMGB1. All structures were preprocessed to remove water molecules and heteroatoms for clear visualization and structural analysis. The respective 3D protein models were then generated, and the selected peptide sequences were superimposed onto them using PyMOL. Visualization confirmed that each peptide was positioned on the solvent-accessible surface of the protein, further validating their potential recognition by circulating antibodies in an immunoassay setting (**Figures 2A–C**). This integration of surface accessibility prediction and structural mapping provides strong evidence supporting the diagnostic relevance of the chosen peptide candidates.

### Homology screening and peptide selection

All shortlisted peptides met the defined thresholds in both BepiPred 2.0 and ABCpred prediction tools, demonstrating consistent identification as linear B-cell epitopes. The selected peptides showed high prediction scores across methods and overlapped with discontinuous (conformational) epitopes identified through ElliPro, reinforcing their structural and immunological relevance. Additionally, all peptides were confirmed to be antigenic using VaxiJen v2.0, indicating their potential to elicit an immune response. Based on this integrative screening approach, three high-confidence linear B-cell epitopes were finalized, each corresponding to one of the target DAMP proteins: HMGB1_150–165_: GSSGMGKGDPKKPRGK; S100A8_42–57_: NSIIDVYHKYSLIKGN; and S100A9_88–103_: SVKLGHPDTLNQGEFK.

The predicted antigenicity scores using VaxiJen v2.0 were as follows: HMGB1 peptide: 1.60; S100A8 peptide: 1.20; and S100A9 peptide: 0.70.

These values indicate strong immunogenic potential, particularly for the HMGB1 and S100A8 peptides, justifying their selection for subsequent peptide synthesis and ELISA-based diagnostic validation.

### Peptide synthesis and ELISA validation

**Figure 3:**
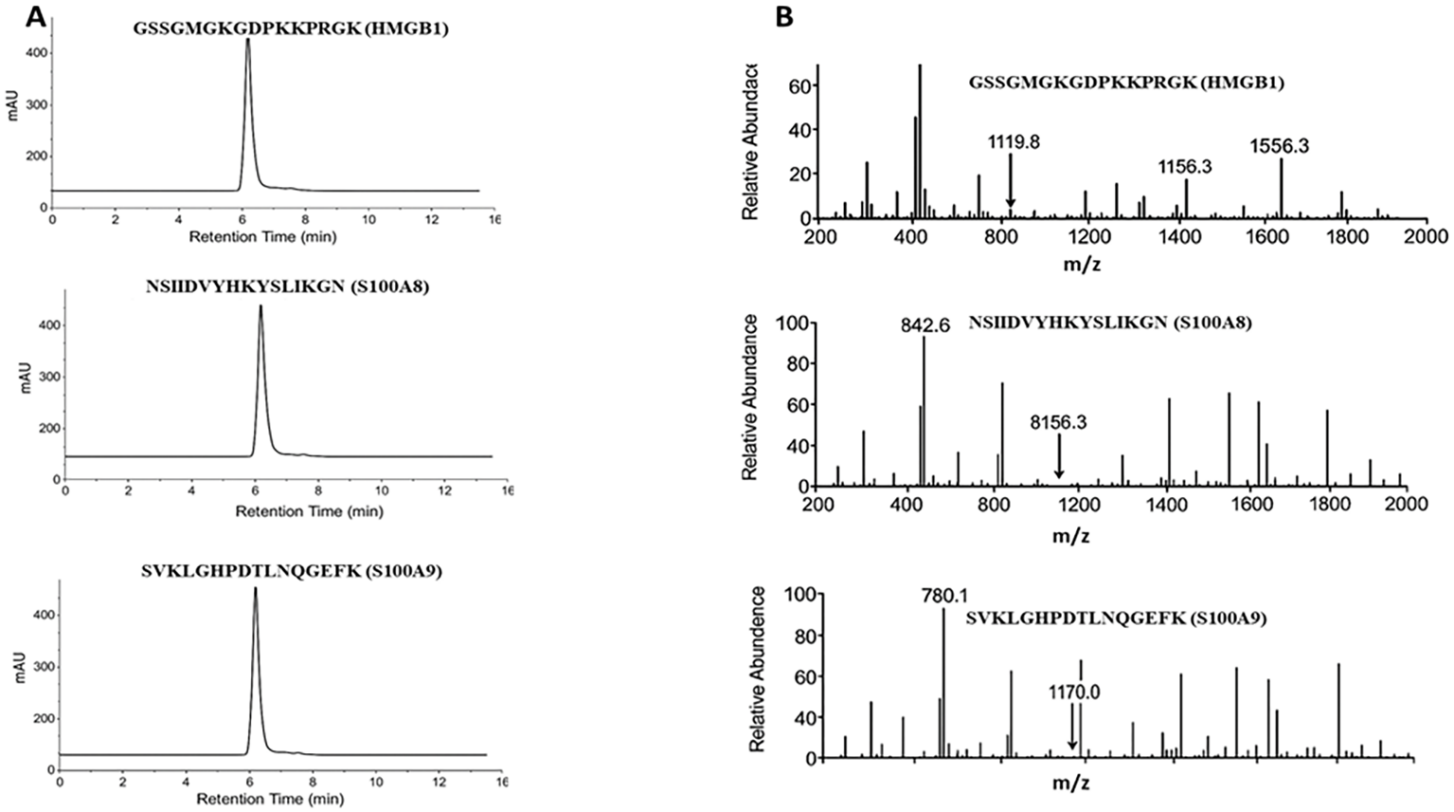
Peptide quality confirmation via HPLC and ESI-MS analyses. **(A)** HPLC chromatograms of synthesized peptides show single major peaks, confirming > 95% purity. **(B)** ESI-MS spectra exhibit m/z peaks corresponding to the theoretical molecular weights of HMGB1, S100A8, and S100A9 peptides.

**Figure 4:**
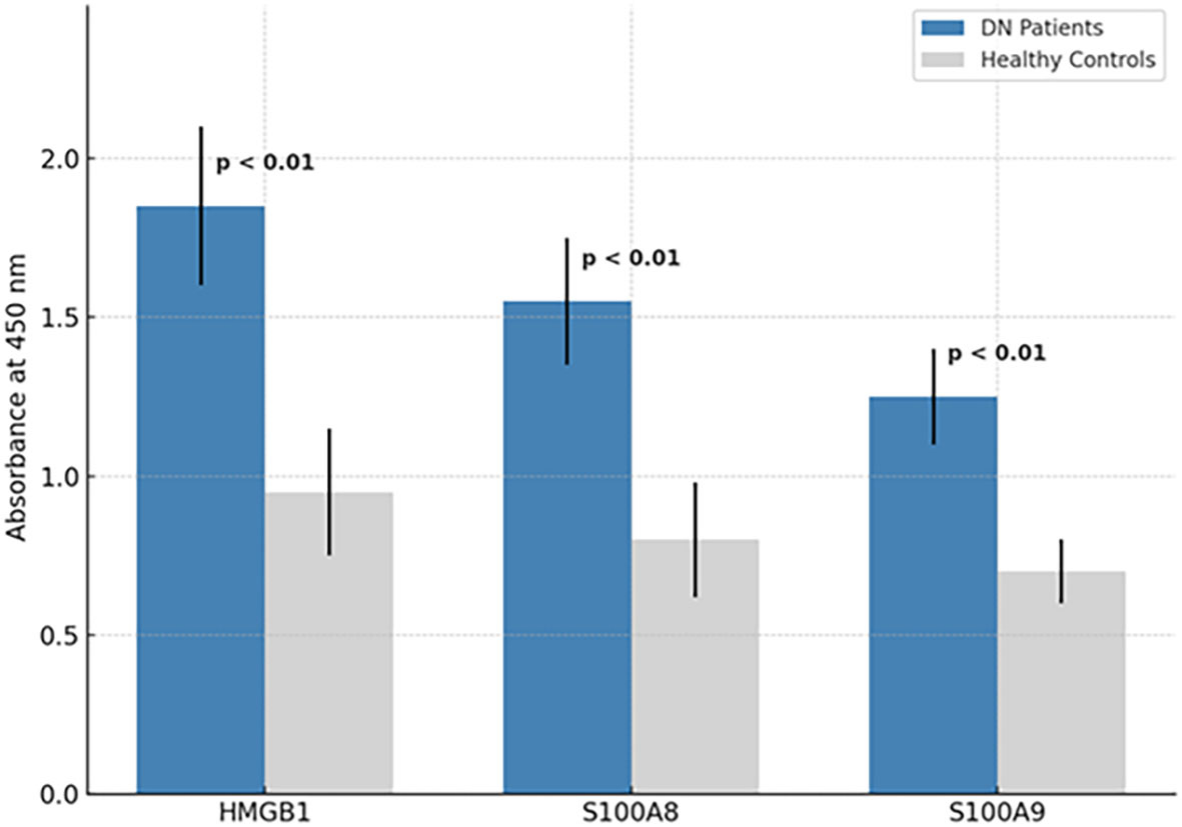
ELISA-based detection of peptide-specific IgG in DN patient and control sera. Indirect ELISA results for HMGB1_150–165_, S100A8_42–57_, and S100A9_88–103_ peptides. Absorbance values at 450 nm show significantly higher IgG binding in DN patient sera (*n* = 30) compared to healthy controls (*n* = 30), with all comparisons yielding *p* < 0.01.

The three selected peptides—GSSGMGKGDPKKPRGK (HMGB1), NSIIDVYHKYSLIKGN (S100A8), and SVKLGHPDTLNQGEFK (S100A9)—were commercially synthesized by a certified peptide manufacturer. To ensure analytical validity, each peptide was accompanied by HPLC and electrospray ionization mass spectrometry (ESI-MS) data. As shown in **Figure 3A** (left panel), all three peptides exhibited single, sharp peaks in their respective HPLC chromatograms, with retention times ranging from 5 to 7 min. The absence of secondary peaks confirmed a purity exceeding 95%, as determined by reverse-phase chromatography, ensuring their suitability for immunological applications such as ELISA. Peptide identity and molecular mass were verified using ESI-MS **Figure 3B**, right panel). The analytical method followed validated procedures for peptide mass accuracy and reproducibility, as described by Hupert et al. (16). The mass spectra revealed prominent m/z peaks consistent with the expected molecular weights: HMGB1 peptide: 1,119.8, 1,156.3, and 1,556.3; S100A8 peptide: 842.6 and 856.3; and S100A9 peptide: 780.1 and 1,170.0 (17, 18). These spectral peaks matched the theoretical fragment masses and isotopic patterns, confirming the structural integrity and correct sequence of each peptide. Collectively, data from the manufacturer demonstrate that the peptides used in this study were of high purity and molecular accuracy, suitable for use in downstream diagnostic validation assays. To evaluate their diagnostic potential, an indirect ELISA was performed using serum samples collected from 30 confirmed DN patients and 30 healthy controls under ethically approved protocols. Each peptide was coated onto ELISA plates at a concentration of 10 µg/mL and incubated with diluted patient and control sera. Peptide-specific IgG antibodies were detected using HRP-conjugated antihuman IgG, followed by colorimetric development with TMB substrate. Absorbance was recorded at 450 nm. The results demonstrated statistically significant elevations in IgG binding in the DN group compared with healthy controls for all three peptides (*p* < 0.01, unpaired *t*-test), suggesting that these peptides are recognized by circulating antibodies in DN patients. Among the three, the HMGB1-derived peptide showed the highest mean absorbance, indicating its superior immunoreactivity and potential as a diagnostic biomarker for DN (**Figure 4**). These findings confirm that the selected peptides are not only structurally and antigenically promising *in silico* but also elicit specific immune recognition in patient samples, validating their use in the development of peptide-based diagnostic assays for diabetic nephropathy.

## Discussion

This study provides a focused, multitiered investigation into the diagnostic potential of epitope-derived peptides from the DAMP proteins HMGB1, S100A8, and S100A9 in DN. By combining advanced immunoinformatics, structural bioinformatics, and experimental serological validation, we identified three novel B-cell epitope peptides that show significant immunoreactivity with DN patient sera. These results represent a critical advance over previous protein-level studies by offering sequence-defined, immunologically active peptide targets suitable for assay development. Several recent studies have highlighted the overexpression and pathological relevance of HMGB1, S100A8, and S100A9 in DN (19). For instance, Chen et al. (20) demonstrated that serum HMGB1 levels correlate with DN stage and renal fibrosis severity. Similarly, Tammaro et al. (21) showed that S100A8 and S100A9 are significantly upregulated in both renal biopsies and urine of DN patients, functioning as amplifiers of sterile inflammation via RAGE and TLR4 signaling. Importantly, several clinical studies have reported that circulating HMGB1 protein levels correlate with DN severity, including associations with reduced eGFR, increased albuminuria, and progression to advanced renal fibrosis, suggesting that HMGB1 biology is linked to both inflammation and structural kidney damage. Thus, correlating peptide-specific IgG titers with established renal indices (eGFR, UACR, serum creatinine, and histological fibrosis scores, where available) will be an essential next step to determine whether the observed humoral responses reflect disease severity or merely the presence/absence of DN (19, 22). Prior studies focused solely on full-protein quantification and did not identify immune-accessible epitopes suitable for targeted diagnostics. Our results address this critical gap by identifying discrete, surface-exposed, and conserved linear peptide segments that are directly recognized by circulating IgG antibodies in DN.

The immunoinformatic strategy used here was designed to optimize both sensitivity and specificity. Consensus-based prediction from BepiPred 2.0 (AUC = 0.85) and ABCpred (AUC = 0.67), each validated in independent benchmark studies (23, 24), enabled robust identification of candidate epitopes. Importantly, we constrained epitope selection to evolutionarily conserved regions across isoforms, a step necessary to minimize polymorphism-induced epitope loss in population-scale diagnostics. This approach addresses a common limitation of prior biomarker studies, where diagnostic accuracy varied across ethnic groups due to underlying genomic variability (25). The top-ranking epitope from HMGB1 (residues 150–165; GSSGMGKGDPKKPRGK) lies within the highly conserved acidic tail domain, previously implicated in DNA binding and cytokine release functions. Consistent with our findings, this region has been reported as surface-accessible in crystallographic studies (26, 27) and shows strong reactivity in rheumatoid arthritis and systemic lupus erythematosus, suggesting its immunodominance in autoimmune contexts (28). The VaxiJen score of 1.60 confirms its strong antigenic potential. Its highest ELISA absorbance among the three peptides in our study further supports this region as a dominant B-cell target in DN. For S100A8, we identified a 16-residue epitope (NSIIDVYHKYSLIKGN) within the calcium-binding EF-hand domain (residues 42–57), a functionally critical region involved in heterodimerization with S100A9 and RAGE activation (29). This matches the region reported by Wang et al. (30), who identified S100A8_42–57_ as a major proinflammatory epitope in glomerulonephritis models. The peptide’s high antigenicity score (1.20) and surface exposure in the 3D model (PDB: 1XK4) underscore its suitability for immune recognition and diagnostic application. Similarly, the S100A9-derived epitope (SVKLGHPDTLNQGEFK) aligns with residues 88–103, a region previously implicated in TLR4-mediated cytokine release in renal ischemia (21). Although its VaxiJen score was lower (0.70), it still exceeded the antigenicity threshold and showed statistically significant ELISA reactivity. Notably, its discontinuous epitope overlap with ElliPro predictions suggests exposure in a conformational context, adding confidence to its immunogenicity. The observed variability in ELISA absorbance for this peptide may reflect interindividual differences in immune recognition or posttranslational masking in native protein conformations.

The use of ElliPro and PyMOL-based 3D mapping provided crucial structural validation. Mapping predicted linear epitopes onto protein crystal structures not only confirmed their surface accessibility but also demonstrated spatial clustering with conformational (discontinuous) epitopes. This dual confirmation has been highlighted as critical in recent B-cell epitope validation frameworks (31), as linear predictions alone often suffer from false positives when buried in folded proteins. The Cα–Cα distance of 4.2 Å ± 0.8 Å for the top HMGB1 epitope suggests tight spatial clustering, a known determinant of antibody affinity (32). The synthesized peptides were confirmed to be > 95% pure via HPLC and consistent with theoretical masses by ESI-MS, ensuring analytical reproducibility. Importantly, indirect ELISA assays revealed significant differences in IgG reactivity between DN patients and healthy controls (*p* < 0.01 for all peptides), indicating disease-specific antibody responses. These results are comparable in magnitude to published reports on peptide ELISA assays in autoimmune nephritis (e.g., anti-C1q and anti-GBM peptide ELISAs), where AUC values typically range from 0.80 to 0.90 (33). While previous studies have used proteomic approaches to identify DN biomarkers (e.g., NGAL, KIM-1), most are renal-tubular or exosome-based and lack the specificity provided by direct immune recognition (34, 35). Moreover, they rely on intact protein detection, which often suffers from instability, batch variability, and posttranslational modification masking. In contrast, peptide-based diagnostics offer advantages in synthetic reproducibility, batch uniformity, and epitope-specific immune detection, aligning with recent trends in autoimmune and infectious disease serodiagnostics (36, 37). By integrating conservation filtering, antigenicity scoring, and structural modeling, our pipeline addresses the key shortcomings of single-tool linear epitope prediction, which often lack precision and real-world translatability. The resulting peptides represent high-confidence targets for further clinical assay development. Importantly, all three peptides demonstrated differential immune recognition in DN patients compared to healthy controls, suggesting their applicability as early detection markers. Given the chronic, subclinical nature of DN, early serological diagnostics could provide a valuable window for intervention before irreversible renal damage occurs.

While this study provides valuable insights into epitope-based diagnostics for DN, several areas merit further investigation to enhance translational relevance. The current ELISA validation, though promising, was limited to a moderate-sized cohort (*n* = 60); future studies will incorporate larger, multicenter cohorts with diverse ethnic representation to establish diagnostic thresholds and generalizability. Additionally, we focused exclusively on IgG isotypes; expanding to IgM and IgA profiling, along with longitudinal sampling, will help clarify isotype dynamics and prognostic relevance. Although *in silico* models confirmed epitope accessibility, validating antibody binding to native full-length proteins remains essential and will be addressed through peptide competition assays and monoclonal antibody mapping. Moreover, while we focused on three key DAMPs, DN pathogenesis involves broader inflammatory networks; thus, future work will extend this platform to additional targets such as RAGE, IL-18, and TGF-β1 to develop a comprehensive, multiplexed diagnostic panel. Although our current validation was limited to serological assessment, correlating DAMP-derived antibody responses with renal biochemical indices such as serum creatinine, blood urea nitrogen (BUN), and urinary protein will be essential to confirm clinical utility. Future studies with expanded patient cohorts will incorporate these parameters to determine whether peptide-specific IgG titers quantitatively reflect renal functional decline and disease progression.

## Conclusion

This study presents a robust immunoinformatics-to-validation pipeline for identifying serologically detectable B-cell epitopes from HMGB1, S100A8, and S100A9—three key DAMP proteins implicated in diabetic nephropathy. By integrating sequence conservation, structural accessibility, and antigenicity prediction with peptide synthesis and ELISA-based validation, we demonstrate that these peptides—particularly the HMGB1_150–165_ fragment—elicit a specific antibody response in DN patient sera. These findings provide a foundation for developing noninvasive, peptide-based diagnostic assays for early DN detection. The approach outlined here can be readily adapted to other inflammatory renal disorders, supporting broader applications in precision nephrology and immunodiagnostics.

## Data Availability

The datasets presented in this study can be found in online repositories. The names of the repository/repositories and accession number(s) can be found in the article/**Supplementary Material**.

## References

[1] Jha R Lopez-Trevino S Kankanamalage HR Jha JC . Diabetes and renal complications: an overview on pathophysiology, biomarkers and therapeutic interventions. Biomedicines. (2024) 12:1098. doi: 10.3390/biomedicines12051098, PMID: 38791060 PMC11118045

[2] Xie P Xie W Wang Z Guo Z Tang R Yang H . Association of diabetic nephropathy with lipid metabolism: a Mendelian randomization study. Diabetol Metab Syndr. (2025) 17:102. doi: 10.1186/s13098-025-01641-8, PMID: 40133980 PMC11938690

[3] Cao Y Lin J-H Hammes H-P Zhang C . Cellular phenotypic transitions in diabetic nephropathy: An update. Front Pharmacol. (2022) 13:1038073. doi: 10.3389/fphar.2022.1038073, PMID: 36408221 PMC9666367

[4] Habli MM . Comprehensive insights into diabetic nephropathy: pathophysiology, clinical features, and emerging treatments. J Egyptian Soc Nephrol Transplant. (2024) 24:163. doi: 10.4103/jesnt.jesnt_16_24

[5] Yamanouchi M Sawa N Toyama T Shimizu M Oshima M Yoshimura Y . Trajectory of GFR decline and fluctuation in albuminuria leading to end-stage kidney disease in patients with biopsy-confirmed diabetic kidney disease. Kidney Int Rep. (2024) 9:323–33. doi: 10.1016/j.ekir.2023.11.004, PMID: 38344735 PMC10851062

[6] Ratan Y Rajput A Pareek A Singh G . Comprehending the role of metabolic and hemodynamic factors alongside different signaling pathways in the pathogenesis of diabetic nephropathy. Int J Mol Sci. (2025) 26:3330. doi: 10.3390/ijms26073330, PMID: 40244213 PMC11989741

[7] Rosin DL Okusa MD . Dangers within: DAMP responses to damage and cell death in kidney disease. J Am Soc Nephrol. (2011) 22:416–25. doi: 10.1681/ASN.2010040430, PMID: 21335516 PMC4493973

[8] Zhang X Zhang J Ren Y Sun R Zhai X . Unveiling the pathogenesis and therapeutic approaches for diabetic nephropathy: insights from panvascular diseases. Front Endocrinol. (2024) 15:1368481. doi: 10.3389/fendo.2024.1368481, PMID: 38455648 PMC10918691

[9] Biscetti F Rando MM Nardella E Cecchini AL Pecorini G Landolfi R . High mobility group box-1 and diabetes mellitus complications: state of the art and future perspectives. Int J Mol Sci. (2019) 20:6258. doi: 10.3390/ijms20246258, PMID: 31835864 PMC6940913

[10] Mukherjee TK Malik P Rani R Mondal H Malik P Rani R . Glycosylation and glycation in health and diseases. Bentham Science Publishers (2025). doi: 10.2174/97898153225211250101

[11] Potocnakova L Bhide M Pulzova LB . An introduction to B-cell epitope mapping and in silico epitope prediction. J Immunol Res. (2016) 2016:6760830. doi: 10.1155/2016/6760830, PMID: 28127568 PMC5227168

[12] De Luca G , Goette NP Lev PR Baroni Pieto MC Marin Oyarzu CP Castro Rios MA . Elevated levels of damage-associated molecular patterns HMGB1 and S100A8/A9 coupled with toll-like receptor-triggered monocyte activation are associated with inflammation in patients with myelofibrosis. Front Immunol. (2024) 15:1365015. doi: 10.3389/fimmu.2024.1365015, PMID: 39391311 PMC11465240

[13] Grewal S Hegde N Yanow SK . Integrating machine learning to advance epitope mapping. Front Immunol. (2024) 15:1463931. doi: 10.3389/fimmu.2024.1463931, PMID: 39403389 PMC11471525

[14] Chen Y Liu X Shengbu M Shi Q Jiaqiu S Lai X . Biomarkers: new advances in diabetic nephropathy. Natural Prod Commun. (2025) 20:1–21. doi: 10.1177/1934578X251321758

[15] Fan C Yang G Li C Cheng J Chen S Mi H . Uncovering glycolysis-driven molecular subtypes in diabetic nephropathy: a WGCNA and machine learning approach for diagnostic precision. Biol Dir. (2025) 20:10. doi: 10.1186/s13062-025-00601-6, PMID: 39838413 PMC11748251

[16] Hupert M Elfgen A Schartmann E Schemmert S Buscher B Kutzsche J . Development and validation of an UHPLC-ESI-QTOF-MS method for quantification of the highly hydrophilic amyloid-β oligomer eliminating all-D-enantiomeric peptide RD2 in mouse plasma. Journal of chromatography. B Anal Technol Biomed Life Sci. (2018) 1073:123–9. doi: 10.1016/j.jchromb.2017.12.009, PMID: 29248770

[17] Zheng Q Dai Z . Engineering HMGB1-derived peptides to unravel sex-specific mechanisms in pulmonary arterial hypertension. Am J Respir Cell Mol Biol. (2025) 73:337–9. doi: 10.1165/rcmb.2025-0070ED, PMID: 40132168 PMC12416314

[18] Wiles TA Saba LM Delong T . Peptide-spectrum match validation with internal standards (P-VIS): internally-controlled validation of mass spectrometry-based peptide identifications. J Proteome Res. (2021) 20:236–49. doi: 10.1021/acs.jproteome.0c00355, PMID: 32924495 PMC7775876

[19] Peng R Zuo S Li X Huang Y Chen S Zou X . Investigating HMGB1 as a potential serum biomarker for early diabetic nephropathy monitoring by quantitative proteomics. iScience. (2024) 27:108834. doi: 10.1016/j.isci.2024.108834, PMID: 38303703 PMC10830865

[20] Chen Y Qiao F Zhao Y Wang Y Liu G . HMGB1 is activated in type 2 diabetes mellitus patients and in mesangial cells in response to high glucose. Int J Clin Exp Pathol. (2015) 8:6683–91. PMC4525884 26261550

[21] Tammaro A Florquin S Brok M Claessen N Butter LM Teske GJD . S100A8/A9 promotes parenchymal damage and renal fibrosis in obstructive nephropathy. Clin Exp Immunol. (2018) 193:361–75. doi: 10.1111/cei.13154, PMID: 29746703 PMC6150262

[22] Liu T Zhao H Wang Y Qu P Wang Y Wu X . Serum high mobility group box 1 as a potential biomarker for the progression of kidney disease in patients with type 2 diabetes. Front Immunol. (2024) 15:1334109. doi: 10.3389/fimmu.2024.1334109, PMID: 38481996 PMC10932975

[23] Saha S Raghava GPS . Prediction of continuous B-cell epitopes in an antigen using recurrent neural network. Proteins. (2006) 65:40–8. doi: 10.1002/prot.21078, PMID: 16894596

[24] Jespersen MC Peters B Nielsen M Marcatili P . BepiPred-2.0: improving sequence-based B-cell epitope prediction using conformational epitopes. Nucleic Acids Res. (2017) 45:W24–9. doi: 10.1093/nar/gkx346, PMID: 28472356 PMC5570230

[25] Zhou J Chen J Peng Y Xie Y Xiao Y . A promising tool in serological diagnosis: current research progress of antigenic epitopes in infectious diseases. Pathogens. (2022) 11:1095. doi: 10.3390/pathogens11101095, PMID: 36297152 PMC9609281

[26] Voong CK Goodrich JA Kugel JF . Interactions of HMGB proteins with the genome and the impact on disease. Biomolecules. (2021) 11:1451. doi: 10.3390/biom11101451, PMID: 34680084 PMC8533419

[27] Chen R Kang R Tang D . The mechanism of HMGB1 secretion and release. Exp Mol Med. (2022) 54:91–102. doi: 10.1038/s12276-022-00736-w, PMID: 35217834 PMC8894452

[28] Pullerits R Jonsson I-M Verdrengh M Bokarewa M Andersson U Erlandsson-Harris H . High mobility group box chromosomal protein 1, a DNA binding cytokine, induces arthritis. Arthritis Rheum. (2003) 48:1693–700. doi: 10.1002/art.11028, PMID: 12794838

[29] Foell D Wittkowski H Vogl T Roth J . S100 proteins expressed in phagocytes: a novel group of damage-associated molecular pattern molecules. J Leukoc Biol. (2007) 81:28–37. doi: 10.1189/jlb.0306170, PMID: 16943388

[30] Wang S Song R Wang Z Jing Z Wang S Ma J . S100A8/A9 in inflammation. Front Immunol. (2018) 9:1298. doi: 10.3389/fimmu.2018.01298, PMID: 29942307 PMC6004386

[31] Zheng D Liang S Zhang C . B-cell epitope predictions using computational methods. Methods Mol Biol. (2023) 2552:239–54. doi: 10.1007/978-1-0716-2609-2_12, PMID: 36346595

[32] Koide H Kiyokawa C Okishima A Saito K Yoshimatsu K Fukuta T . Design of an anti-HMGB1 synthetic antibody for *in vivo* ischemic/reperfusion injury therapy. J Am Chem Soc. (2023) 145:23143–51. doi: 10.1021/jacs.3c06799, PMID: 37844138 PMC10603801

[33] Sun Y Fong K-Y Chung MCM Yao Z-J . Peptide mimicking antigenic and immunogenic epitope of double-stranded DNA in systemic lupus erythematosus. Int Immunol. (2001) 13:223–32. doi: 10.1093/intimm/13.2.223, PMID: 11157855

[34] Obert LA Elmore SA Ennulat D Frazier KS . A review of specific biomarkers of chronic renal injury and their potential application in nonclinical safety assessment studies. Toxicol Pathol. (2021) 49:996–1023. doi: 10.1177/0192623320985045, PMID: 33576319 PMC8195817

[35] Rico-Fontalvo J Aroca-Martínez G Daza-Arnedo R Cabrales J Rodríguez-Yanez T Cardona-Blanco M . Novel biomarkers of diabetic kidney disease. Biomolecules. (2023) 13:633. doi: 10.3390/biom13040633, PMID: 37189380 PMC10135955

[36] Gómara MJ Haro I . Synthetic peptides for the immunodiagnosis of human diseases. Curr Med Chem. (2007) 14:531–46. doi: 10.2174/092986707780059698, PMID: 17346145

[37] Pandey S Malviya G Chottova Dvorakova M . Role of peptides in diagnostics. Int J Mol Sci. (2021) 22:8828. doi: 10.3390/ijms22168828, PMID: 34445532 PMC8396325

