# Supplementary material for: Discovery and serological validation of DAMP-derived B-cell epitopes as diagnostic biomarkers for diabetic nephropathy
Source: Front Endocrinol (Lausanne). 2025 Nov 24;16:1652922. doi: 10.3389/fendo.2025.1652922 (PMC12682578; doi:10.3389/fendo.2025.1652922)
Supplement: Supplementary file 1 [file DataSheet1.docx]

Supplimentory file


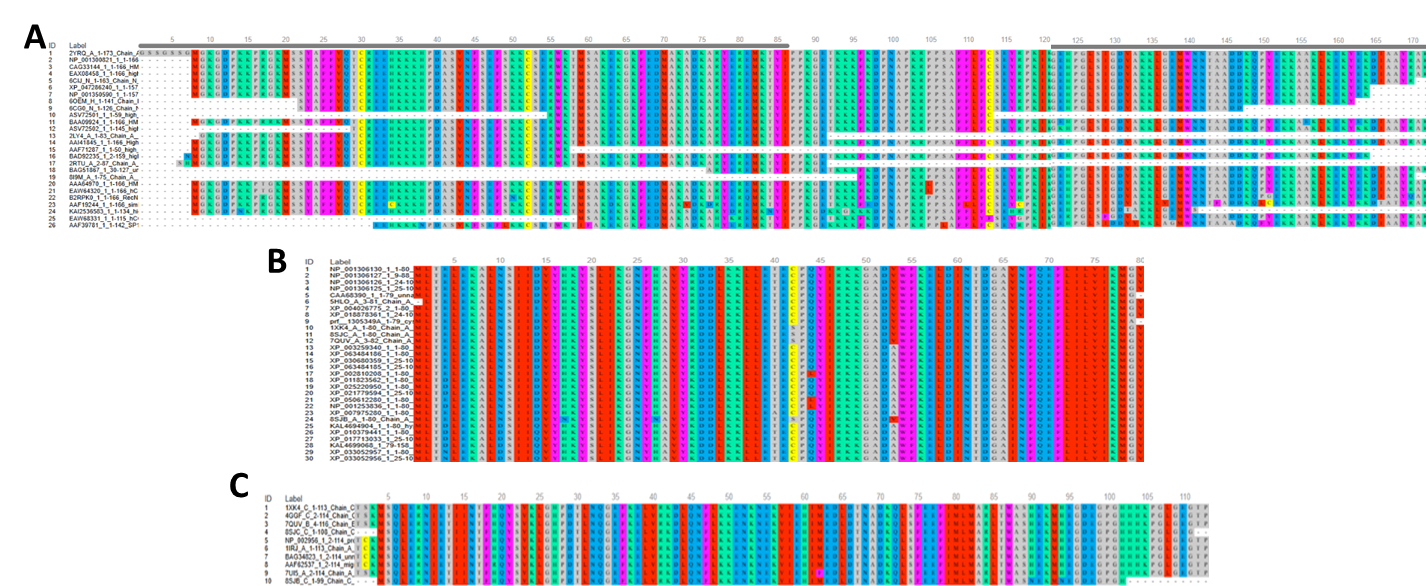


**Supplementary Figure 1:** Multiple sequence alignments of the target proteins using MAFFTv.7 server.

A: HMGB-1, B: S100A8, and C: S100A9

**Supplementary table 1 :** The epitopes predicted by Bepipred server for HMGB-1 protein.

| Entry | Position | AminoAcid | Exposed/Buried | RelativeSurfaceAccessilibity | HelixProbability | SheetProbability | CoilProbability | EpitopeProbability |
| --- | --- | --- | --- | --- | --- | --- | --- | --- |
| Sequence | 1 | G | E | 0.829 | 0.003 | 0.003 | 0.994 | 0.240444 |
| Sequence | 2 | S | E | 0.594 | 0.058 | 0.017 | 0.925 | 0.315444 |
| Sequence | 3 | S | E | 0.521 | 0.113 | 0.043 | 0.844 | 0.385 |
| Sequence | 4 | G | E | 0.419 | 0.113 | 0.043 | 0.844 | 0.450778 |
| Sequence | 5 | S | E | 0.445 | 0.184 | 0.043 | 0.773 | 0.512222 |
| Sequence | 6 | S | E | 0.386 | 0.184 | 0.043 | 0.773 | 0.550889 |
| Sequence | 7 | G | B | 0.206 | 0.278 | 0.093 | 0.628 | 0.571222 |
| Sequence | 8 | M | E | 0.257 | 0.278 | 0.093 | 0.628 | 0.582778 |
| Sequence | 9 | G | B | 0.148 | 0.386 | 0.097 | 0.517 | 0.575556 |
| Sequence | 10 | K | B | 0.149 | 0.386 | 0.097 | 0.517 | 0.573111 |
| Sequence | 11 | G | B | 0.128 | 0.386 | 0.097 | 0.517 | 0.556444 |
| Sequence | 12 | D | B | 0.111 | 0.386 | 0.097 | 0.517 | 0.537222 |
| Sequence | 13 | P | B | 0.06 | 0.386 | 0.097 | 0.517 | 0.527222 |
| Sequence | 14 | K | B | 0.132 | 0.386 | 0.097 | 0.517 | 0.518 |
| Sequence | 15 | K | B | 0.151 | 0.354 | 0.048 | 0.598 | 0.509222 |
| Sequence | 16 | P | B | 0.081 | 0.455 | 0.046 | 0.498 | 0.510889 |
| Sequence | 17 | R | B | 0.121 | 0.354 | 0.048 | 0.598 | 0.517778 |
| Sequence | 18 | G | B | 0.134 | 0.354 | 0.048 | 0.598 | 0.524778 |
| Sequence | 19 | K | B | 0.183 | 0.268 | 0.043 | 0.689 | 0.516222 |
| Sequence | 20 | M | B | 0.237 | 0.053 | 0.043 | 0.903 | 0.515444 |
| Sequence | 21 | S | E | 0.308 | 0.018 | 0.019 | 0.964 | 0.517444 |
| Sequence | 22 | S | E | 0.378 | 0.018 | 0.019 | 0.964 | 0.509333 |
| Sequence | 23 | Y | B | 0.197 | 0.018 | 0.019 | 0.964 | 0.508333 |
| Sequence | 24 | A | B | 0.167 | 0.018 | 0.047 | 0.935 | 0.501222 |
| Sequence | 25 | F | B | 0.123 | 0.052 | 0.084 | 0.864 | 0.486667 |
| Sequence | 26 | F | B | 0.079 | 0.056 | 0.142 | 0.802 | 0.464556 |
| Sequence | 27 | V | B | 0.087 | 0.113 | 0.087 | 0.8 | 0.452556 |
| Sequence | 28 | Q | B | 0.1 | 0.522 | 0.016 | 0.462 | 0.45 |
| Sequence | 29 | T | B | 0.142 | 0.502 | 0.002 | 0.495 | 0.448444 |
| Sequence | 30 | C | B | 0.121 | 0.6 | 0.003 | 0.397 | 0.453333 |
| Sequence | 31 | R | B | 0.088 | 0.6 | 0.003 | 0.397 | 0.461444 |
| Sequence | 32 | E | B | 0.138 | 0.622 | 0.015 | 0.363 | 0.469222 |
| Sequence | 33 | E | B | 0.129 | 0.522 | 0.016 | 0.462 | 0.483 |
| Sequence | 34 | H | B | 0.181 | 0.339 | 0.016 | 0.645 | 0.493444 |
| Sequence | 35 | K | B | 0.146 | 0.268 | 0.043 | 0.689 | 0.499333 |
| Sequence | 36 | K | B | 0.147 | 0.268 | 0.043 | 0.689 | 0.506667 |
| Sequence | 37 | K | B | 0.144 | 0.053 | 0.043 | 0.903 | 0.498889 |
| Sequence | 38 | H | E | 0.241 | 0.018 | 0.047 | 0.935 | 0.501667 |
| Sequence | 39 | P | B | 0.053 | 0.018 | 0.047 | 0.935 | 0.500222 |
| Sequence | 40 | D | B | 0.115 | 0.018 | 0.088 | 0.893 | 0.501222 |
| Sequence | 41 | A | B | 0.107 | 0.018 | 0.047 | 0.935 | 0.487889 |
| Sequence | 42 | S | E | 0.23 | 0.018 | 0.088 | 0.893 | 0.477 |
| Sequence | 43 | V | B | 0.104 | 0.052 | 0.084 | 0.864 | 0.482889 |
| Sequence | 44 | N | B | 0.209 | 0.052 | 0.084 | 0.864 | 0.483778 |
| Sequence | 45 | F | B | 0.091 | 0.052 | 0.084 | 0.864 | 0.486111 |
| Sequence | 46 | S | E | 0.247 | 0.052 | 0.084 | 0.864 | 0.499889 |
| Sequence | 47 | E | B | 0.139 | 0.052 | 0.084 | 0.864 | 0.506111 |
| Sequence | 48 | F | B | 0.085 | 0.053 | 0.043 | 0.903 | 0.503889 |
| Sequence | 49 | S | B | 0.218 | 0.113 | 0.087 | 0.8 | 0.504778 |
| Sequence | 50 | K | B | 0.181 | 0.184 | 0.043 | 0.773 | 0.517222 |
| Sequence | 51 | K | B | 0.13 | 0.115 | 0.016 | 0.868 | 0.520444 |
| Sequence | 52 | C | B | 0.149 | 0.181 | 0.016 | 0.803 | 0.518111 |
| Sequence | 53 | S | E | 0.316 | 0.257 | 0.016 | 0.727 | 0.530111 |
| Sequence | 54 | E | B | 0.207 | 0.257 | 0.016 | 0.727 | 0.525778 |
| Sequence | 55 | R | B | 0.106 | 0.184 | 0.043 | 0.773 | 0.528778 |
| Sequence | 56 | W | B | 0.123 | 0.184 | 0.043 | 0.773 | 0.523667 |
| Sequence | 57 | K | B | 0.131 | 0.184 | 0.043 | 0.773 | 0.528 |
| Sequence | 58 | T | B | 0.213 | 0.184 | 0.043 | 0.773 | 0.529333 |
| Sequence | 59 | M | B | 0.223 | 0.354 | 0.048 | 0.598 | 0.528111 |
| Sequence | 60 | S | E | 0.309 | 0.455 | 0.046 | 0.498 | 0.530556 |
| Sequence | 61 | A | B | 0.181 | 0.43 | 0.016 | 0.555 | 0.523889 |
| Sequence | 62 | K | B | 0.126 | 0.43 | 0.016 | 0.555 | 0.518222 |
| Sequence | 63 | E | B | 0.126 | 0.522 | 0.016 | 0.462 | 0.514556 |
| Sequence | 64 | K | B | 0.133 | 0.561 | 0.047 | 0.393 | 0.506889 |
| Sequence | 65 | G | B | 0.098 | 0.455 | 0.046 | 0.498 | 0.503333 |
| Sequence | 66 | K | B | 0.14 | 0.455 | 0.046 | 0.498 | 0.500667 |
| Sequence | 67 | F | B | 0.1 | 0.354 | 0.048 | 0.598 | 0.495111 |
| Sequence | 68 | E | B | 0.112 | 0.268 | 0.043 | 0.689 | 0.485889 |
| Sequence | 69 | D | B | 0.134 | 0.278 | 0.093 | 0.628 | 0.476111 |
| Sequence | 70 | M | B | 0.14 | 0.191 | 0.086 | 0.723 | 0.472111 |
| Sequence | 71 | A | B | 0.14 | 0.278 | 0.093 | 0.628 | 0.466889 |
| Sequence | 72 | K | B | 0.098 | 0.455 | 0.046 | 0.498 | 0.467556 |
| Sequence | 73 | A | B | 0.1 | 0.43 | 0.016 | 0.555 | 0.465667 |
| Sequence | 74 | D | B | 0.074 | 0.622 | 0.015 | 0.363 | 0.458111 |
| Sequence | 75 | K | B | 0.086 | 0.717 | 0.014 | 0.269 | 0.449 |
| Sequence | 76 | A | B | 0.104 | 0.717 | 0.014 | 0.269 | 0.450333 |
| Sequence | 77 | R | B | 0.061 | 0.717 | 0.014 | 0.269 | 0.462111 |
| Sequence | 78 | Y | B | 0.119 | 0.622 | 0.015 | 0.363 | 0.463778 |
| Sequence | 79 | E | B | 0.098 | 0.622 | 0.015 | 0.363 | 0.468778 |
| Sequence | 80 | R | B | 0.085 | 0.522 | 0.016 | 0.462 | 0.466889 |
| Sequence | 81 | E | B | 0.127 | 0.43 | 0.016 | 0.555 | 0.470778 |
| Sequence | 82 | M | B | 0.164 | 0.339 | 0.016 | 0.645 | 0.466778 |
| Sequence | 83 | K | B | 0.081 | 0.181 | 0.016 | 0.803 | 0.463333 |
| Sequence | 84 | T | B | 0.151 | 0.113 | 0.043 | 0.844 | 0.456222 |
| Sequence | 85 | Y | B | 0.108 | 0.191 | 0.086 | 0.723 | 0.448556 |
| Sequence | 86 | I | B | 0.07 | 0.278 | 0.093 | 0.628 | 0.432222 |
| Sequence | 87 | P | B | 0.045 | 0.268 | 0.043 | 0.689 | 0.435111 |
| Sequence | 88 | P | B | 0.052 | 0.455 | 0.046 | 0.498 | 0.432889 |
| Sequence | 89 | K | B | 0.084 | 0.522 | 0.016 | 0.462 | 0.439444 |
| Sequence | 90 | G | B | 0.065 | 0.561 | 0.047 | 0.393 | 0.442 |
| Sequence | 91 | E | B | 0.065 | 0.561 | 0.047 | 0.393 | 0.450222 |
| Sequence | 92 | T | B | 0.119 | 0.455 | 0.046 | 0.498 | 0.451111 |
| Sequence | 93 | K | B | 0.108 | 0.455 | 0.046 | 0.498 | 0.463778 |
| Sequence | 94 | K | B | 0.12 | 0.455 | 0.046 | 0.498 | 0.471667 |
| Sequence | 95 | K | B | 0.118 | 0.354 | 0.048 | 0.598 | 0.483 |
| Sequence | 96 | F | B | 0.099 | 0.354 | 0.048 | 0.598 | 0.486444 |
| Sequence | 97 | K | B | 0.109 | 0.268 | 0.043 | 0.689 | 0.477111 |
| Sequence | 98 | D | B | 0.113 | 0.191 | 0.086 | 0.723 | 0.466222 |
| Sequence | 99 | P | B | 0.054 | 0.191 | 0.086 | 0.723 | 0.466778 |
| Sequence | 100 | N | E | 0.235 | 0.191 | 0.086 | 0.723 | 0.460889 |
| Sequence | 101 | A | B | 0.118 | 0.191 | 0.086 | 0.723 | 0.467 |
| Sequence | 102 | P | B | 0.045 | 0.184 | 0.043 | 0.773 | 0.460111 |
| Sequence | 103 | K | B | 0.124 | 0.184 | 0.043 | 0.773 | 0.456333 |
| Sequence | 104 | R | B | 0.104 | 0.113 | 0.043 | 0.844 | 0.451778 |
| Sequence | 105 | P | B | 0.068 | 0.113 | 0.043 | 0.844 | 0.446556 |
| Sequence | 106 | P | B | 0.065 | 0.053 | 0.043 | 0.903 | 0.451444 |
| Sequence | 107 | S | B | 0.224 | 0.018 | 0.019 | 0.964 | 0.456222 |
| Sequence | 108 | A | B | 0.153 | 0.018 | 0.047 | 0.935 | 0.451 |
| Sequence | 109 | F | B | 0.107 | 0.053 | 0.043 | 0.903 | 0.449333 |
| Sequence | 110 | F | B | 0.093 | 0.056 | 0.142 | 0.802 | 0.449222 |
| Sequence | 111 | L | B | 0.096 | 0.056 | 0.142 | 0.802 | 0.460222 |
| Sequence | 112 | F | B | 0.099 | 0.052 | 0.084 | 0.864 | 0.467556 |
| Sequence | 113 | C | B | 0.11 | 0.113 | 0.043 | 0.844 | 0.469667 |
| Sequence | 114 | S | B | 0.205 | 0.339 | 0.016 | 0.645 | 0.472 |
| Sequence | 115 | E | B | 0.117 | 0.339 | 0.016 | 0.645 | 0.476222 |
| Sequence | 116 | Y | B | 0.091 | 0.43 | 0.016 | 0.555 | 0.470333 |
| Sequence | 117 | R | B | 0.047 | 0.43 | 0.016 | 0.555 | 0.473778 |
| Sequence | 118 | P | B | 0.052 | 0.455 | 0.046 | 0.498 | 0.473889 |
| Sequence | 119 | K | B | 0.074 | 0.455 | 0.046 | 0.498 | 0.478556 |
| Sequence | 120 | I | B | 0.101 | 0.455 | 0.046 | 0.498 | 0.479556 |
| Sequence | 121 | K | B | 0.126 | 0.354 | 0.048 | 0.598 | 0.478 |
| Sequence | 122 | G | B | 0.107 | 0.268 | 0.043 | 0.689 | 0.476556 |
| Sequence | 123 | E | B | 0.134 | 0.113 | 0.043 | 0.844 | 0.477111 |
| Sequence | 124 | H | B | 0.229 | 0.018 | 0.047 | 0.935 | 0.482222 |
| Sequence | 125 | P | B | 0.068 | 0.052 | 0.084 | 0.864 | 0.486222 |
| Sequence | 126 | G | B | 0.108 | 0.056 | 0.142 | 0.802 | 0.480667 |
| Sequence | 127 | L | B | 0.104 | 0.056 | 0.142 | 0.802 | 0.484778 |
| Sequence | 128 | S | B | 0.238 | 0.052 | 0.084 | 0.864 | 0.481778 |
| Sequence | 129 | I | B | 0.106 | 0.113 | 0.087 | 0.8 | 0.481222 |
| Sequence | 130 | G | B | 0.131 | 0.118 | 0.15 | 0.732 | 0.485556 |
| Sequence | 131 | D | B | 0.13 | 0.199 | 0.152 | 0.649 | 0.496222 |
| Sequence | 132 | V | B | 0.109 | 0.191 | 0.086 | 0.723 | 0.494667 |
| Sequence | 133 | A | B | 0.126 | 0.278 | 0.093 | 0.628 | 0.485667 |
| Sequence | 134 | K | B | 0.158 | 0.278 | 0.093 | 0.628 | 0.494222 |
| Sequence | 135 | K | B | 0.146 | 0.268 | 0.043 | 0.689 | 0.496889 |
| Sequence | 136 | L | B | 0.107 | 0.278 | 0.093 | 0.628 | 0.507667 |
| Sequence | 137 | G | B | 0.108 | 0.278 | 0.093 | 0.628 | 0.519333 |
| Sequence | 138 | E | B | 0.175 | 0.199 | 0.152 | 0.649 | 0.528889 |
| Sequence | 139 | M | B | 0.24 | 0.118 | 0.15 | 0.732 | 0.529889 |
| Sequence | 140 | W | B | 0.112 | 0.118 | 0.15 | 0.732 | 0.527 |
| Sequence | 141 | N | E | 0.274 | 0.118 | 0.15 | 0.732 | 0.531111 |
| Sequence | 142 | N | B | 0.257 | 0.052 | 0.084 | 0.864 | 0.539778 |
| Sequence | 143 | T | B | 0.201 | 0.113 | 0.087 | 0.8 | 0.542667 |
| Sequence | 144 | A | B | 0.179 | 0.191 | 0.086 | 0.723 | 0.551444 |
| Sequence | 145 | A | B | 0.183 | 0.268 | 0.043 | 0.689 | 0.544 |
| Sequence | 146 | D | B | 0.084 | 0.268 | 0.043 | 0.689 | 0.526889 |
| Sequence | 147 | D | B | 0.066 | 0.354 | 0.048 | 0.598 | 0.508556 |
| Sequence | 148 | K | B | 0.081 | 0.455 | 0.046 | 0.498 | 0.488889 |
| Sequence | 149 | Q | B | 0.09 | 0.455 | 0.046 | 0.498 | 0.479667 |
| Sequence | 150 | P | B | 0.039 | 0.455 | 0.046 | 0.498 | 0.472667 |
| Sequence | 151 | Y | B | 0.075 | 0.455 | 0.046 | 0.498 | 0.455444 |
| Sequence | 152 | E | B | 0.063 | 0.561 | 0.047 | 0.393 | 0.438222 |
| Sequence | 153 | K | B | 0.059 | 0.622 | 0.015 | 0.363 | 0.415778 |
| Sequence | 154 | K | B | 0.053 | 0.622 | 0.015 | 0.363 | 0.400444 |
| Sequence | 155 | A | B | 0.059 | 0.717 | 0.014 | 0.269 | 0.399222 |
| Sequence | 156 | A | B | 0.081 | 0.802 | 0.014 | 0.185 | 0.404 |
| Sequence | 157 | K | B | 0.066 | 0.717 | 0.014 | 0.269 | 0.412222 |
| Sequence | 158 | L | B | 0.066 | 0.717 | 0.014 | 0.269 | 0.416778 |
| Sequence | 159 | K | B | 0.086 | 0.717 | 0.014 | 0.269 | 0.412889 |
| Sequence | 160 | E | B | 0.103 | 0.622 | 0.015 | 0.363 | 0.426444 |
| Sequence | 161 | K | B | 0.097 | 0.43 | 0.016 | 0.555 | 0.441444 |
| Sequence | 162 | Y | B | 0.13 | 0.339 | 0.016 | 0.645 | 0.449 |
| Sequence | 163 | E | B | 0.081 | 0.268 | 0.043 | 0.689 | 0.457111 |
| Sequence | 164 | K | B | 0.082 | 0.184 | 0.043 | 0.773 | 0.457111 |
| Sequence | 165 | D | B | 0.115 | 0.113 | 0.087 | 0.8 | 0.463333 |
| Sequence | 166 | I | B | 0.095 | 0.113 | 0.087 | 0.8 | 0.472222 |
| Sequence | 167 | A | B | 0.177 | 0.113 | 0.043 | 0.844 | 0.477444 |
| Sequence | 168 | A | B | 0.243 | 0.115 | 0.016 | 0.868 | 0.496111 |
| Sequence | 169 | Y | B | 0.233 | 0.184 | 0.043 | 0.773 | 0.504889 |
| Sequence | 170 | R | B | 0.207 | 0.113 | 0.043 | 0.844 | 0.447778 |
| Sequence | 171 | A | E | 0.385 | 0.053 | 0.043 | 0.903 | 0.401444 |
| Sequence | 172 | K | E | 0.426 | 0.053 | 0.043 | 0.903 | 0.350222 |
| Sequence | 173 | G | E | 0.773 | 0.003 | 0.003 | 0.994 | 0.303 |

**Supplementary table 2:** The epitopes predicted by Bepipred server for S100A8 protein.

| Entry | Position | AminoAcid | Exposed/Buried | RelativeSurfaceAccessilibity | HelixProbability | SheetProbability | CoilProbability | EpitopeProbability |
| --- | --- | --- | --- | --- | --- | --- | --- | --- |
| 1 | 1 | M | E | 0.814 | 0.003 | 0.003 | 0.994 | 0.236556 |
| 1 | 2 | L | E | 0.408 | 0.053 | 0.043 | 0.903 | 0.313778 |
| 1 | 3 | T | E | 0.508 | 0.113 | 0.043 | 0.844 | 0.396444 |
| 1 | 4 | E | E | 0.411 | 0.184 | 0.043 | 0.773 | 0.461333 |
| 1 | 5 | L | E | 0.29 | 0.184 | 0.043 | 0.773 | 0.521889 |
| 1 | 6 | E | E | 0.339 | 0.184 | 0.043 | 0.773 | 0.559556 |
| 1 | 7 | K | E | 0.368 | 0.191 | 0.086 | 0.723 | 0.581333 |
| 1 | 8 | A | E | 0.34 | 0.184 | 0.043 | 0.773 | 0.581444 |
| 1 | 9 | L | B | 0.198 | 0.184 | 0.043 | 0.773 | 0.581778 |
| 1 | 10 | N | E | 0.342 | 0.113 | 0.043 | 0.844 | 0.587444 |
| 1 | 11 | S | E | 0.385 | 0.113 | 0.087 | 0.8 | 0.567667 |
| 1 | 12 | I | B | 0.13 | 0.118 | 0.15 | 0.732 | 0.555 |
| 1 | 13 | I | B | 0.146 | 0.118 | 0.15 | 0.732 | 0.561111 |
| 1 | 14 | D | B | 0.191 | 0.118 | 0.15 | 0.732 | 0.561889 |
| 1 | 15 | V | B | 0.188 | 0.113 | 0.087 | 0.8 | 0.548889 |
| 1 | 16 | Y | B | 0.226 | 0.113 | 0.087 | 0.8 | 0.544444 |
| 1 | 17 | H | E | 0.324 | 0.053 | 0.043 | 0.903 | 0.545333 |
| 1 | 18 | K | B | 0.168 | 0.052 | 0.084 | 0.864 | 0.538111 |
| 1 | 19 | Y | B | 0.168 | 0.052 | 0.084 | 0.864 | 0.536444 |
| 1 | 20 | S | B | 0.229 | 0.118 | 0.15 | 0.732 | 0.529889 |
| 1 | 21 | L | B | 0.123 | 0.118 | 0.15 | 0.732 | 0.515222 |
| 1 | 22 | I | B | 0.101 | 0.191 | 0.086 | 0.723 | 0.495667 |
| 1 | 23 | K | B | 0.173 | 0.199 | 0.152 | 0.649 | 0.492667 |
| 1 | 24 | G | B | 0.145 | 0.113 | 0.087 | 0.8 | 0.495778 |
| 1 | 25 | N | E | 0.256 | 0.056 | 0.142 | 0.802 | 0.493333 |
| 1 | 26 | F | B | 0.128 | 0.056 | 0.142 | 0.802 | 0.499333 |
| 1 | 27 | H | B | 0.21 | 0.118 | 0.15 | 0.732 | 0.504222 |
| 1 | 28 | A | B | 0.179 | 0.118 | 0.15 | 0.732 | 0.502667 |
| 1 | 29 | V | B | 0.142 | 0.199 | 0.152 | 0.649 | 0.511 |
| 1 | 30 | Y | B | 0.175 | 0.278 | 0.093 | 0.628 | 0.509444 |
| 1 | 31 | R | B | 0.089 | 0.354 | 0.048 | 0.598 | 0.514 |
| 1 | 32 | D | B | 0.137 | 0.354 | 0.048 | 0.598 | 0.513111 |
| 1 | 33 | D | B | 0.111 | 0.386 | 0.097 | 0.517 | 0.507556 |
| 1 | 34 | L | B | 0.109 | 0.386 | 0.097 | 0.517 | 0.51 |
| 1 | 35 | K | B | 0.156 | 0.386 | 0.097 | 0.517 | 0.506889 |
| 1 | 36 | K | B | 0.171 | 0.386 | 0.097 | 0.517 | 0.504556 |
| 1 | 37 | L | B | 0.144 | 0.386 | 0.097 | 0.517 | 0.500556 |
| 1 | 38 | L | B | 0.145 | 0.278 | 0.093 | 0.628 | 0.487222 |
| 1 | 39 | E | B | 0.16 | 0.191 | 0.086 | 0.723 | 0.486444 |
| 1 | 40 | T | B | 0.23 | 0.113 | 0.043 | 0.844 | 0.485889 |
| 1 | 41 | E | B | 0.198 | 0.113 | 0.043 | 0.844 | 0.487444 |
| 1 | 42 | C | B | 0.168 | 0.113 | 0.043 | 0.844 | 0.482444 |
| 1 | 43 | P | B | 0.075 | 0.268 | 0.043 | 0.689 | 0.486 |
| 1 | 44 | Q | B | 0.233 | 0.455 | 0.046 | 0.498 | 0.490111 |
| 1 | 45 | Y | B | 0.161 | 0.455 | 0.046 | 0.498 | 0.497111 |
| 1 | 46 | I | B | 0.091 | 0.455 | 0.046 | 0.498 | 0.494667 |
| 1 | 47 | R | B | 0.11 | 0.354 | 0.048 | 0.598 | 0.498778 |
| 1 | 48 | K | B | 0.182 | 0.386 | 0.097 | 0.517 | 0.504778 |
| 1 | 49 | K | B | 0.2 | 0.386 | 0.097 | 0.517 | 0.513667 |
| 1 | 50 | G | B | 0.117 | 0.191 | 0.086 | 0.723 | 0.516778 |
| 1 | 51 | A | B | 0.211 | 0.113 | 0.087 | 0.8 | 0.521333 |
| 1 | 52 | D | B | 0.224 | 0.113 | 0.087 | 0.8 | 0.527889 |
| 1 | 53 | V | B | 0.19 | 0.118 | 0.15 | 0.732 | 0.526667 |
| 1 | 54 | W | B | 0.181 | 0.125 | 0.227 | 0.648 | 0.517889 |
| 1 | 55 | F | B | 0.145 | 0.125 | 0.227 | 0.648 | 0.527222 |
| 1 | 56 | K | B | 0.17 | 0.118 | 0.15 | 0.732 | 0.540333 |
| 1 | 57 | E | B | 0.175 | 0.056 | 0.142 | 0.802 | 0.546 |
| 1 | 58 | L | B | 0.156 | 0.066 | 0.296 | 0.638 | 0.533 |
| 1 | 59 | D | B | 0.177 | 0.066 | 0.296 | 0.638 | 0.538444 |
| 1 | 60 | I | B | 0.151 | 0.064 | 0.216 | 0.721 | 0.537556 |
| 1 | 61 | N | E | 0.308 | 0.056 | 0.142 | 0.802 | 0.535444 |
| 1 | 62 | T | B | 0.264 | 0.056 | 0.142 | 0.802 | 0.541111 |
| 1 | 63 | D | B | 0.201 | 0.052 | 0.084 | 0.864 | 0.556 |
| 1 | 64 | G | B | 0.179 | 0.113 | 0.087 | 0.8 | 0.549778 |
| 1 | 65 | A | B | 0.256 | 0.118 | 0.15 | 0.732 | 0.551667 |
| 1 | 66 | V | B | 0.184 | 0.118 | 0.15 | 0.732 | 0.548556 |
| 1 | 67 | N | E | 0.316 | 0.199 | 0.152 | 0.649 | 0.552667 |
| 1 | 68 | F | B | 0.206 | 0.118 | 0.15 | 0.732 | 0.545889 |
| 1 | 69 | Q | B | 0.207 | 0.199 | 0.152 | 0.649 | 0.552667 |
| 1 | 70 | E | B | 0.215 | 0.118 | 0.15 | 0.732 | 0.552667 |
| 1 | 71 | F | B | 0.176 | 0.118 | 0.15 | 0.732 | 0.541667 |
| 1 | 72 | L | B | 0.181 | 0.216 | 0.235 | 0.548 | 0.539778 |
| 1 | 73 | I | B | 0.21 | 0.216 | 0.235 | 0.548 | 0.547 |
| 1 | 74 | L | B | 0.229 | 0.199 | 0.152 | 0.649 | 0.543222 |
| 1 | 75 | V | B | 0.276 | 0.199 | 0.152 | 0.649 | 0.550222 |
| 1 | 76 | I | E | 0.305 | 0.199 | 0.152 | 0.649 | 0.542556 |
| 1 | 77 | K | E | 0.377 | 0.113 | 0.087 | 0.8 | 0.481778 |
| 1 | 78 | M | E | 0.49 | 0.113 | 0.087 | 0.8 | 0.422222 |
| 1 | 79 | G | E | 0.531 | 0.053 | 0.043 | 0.903 | 0.360778 |
| 1 | 80 | V | E | 0.746 | 0.003 | 0.003 | 0.994 | 0.305778 |

**Supplementary table 3:** The epitopes predicted by Bepipred server for S100A9 protein.

| Entry | Position | AminoAcid | Exposed/Buried | RelativeSurfaceAccessilibity | HelixProbability | SheetProbability | CoilProbability | EpitopeProbability |
| --- | --- | --- | --- | --- | --- | --- | --- | --- |
| 1 | 1 | T | E | 0.828 | 0.003 | 0.003 | 0.994 | 0.247778 |
| 1 | 2 | S | E | 0.582 | 0.115 | 0.016 | 0.868 | 0.320778 |
| 1 | 3 | K | E | 0.43 | 0.181 | 0.016 | 0.803 | 0.379444 |
| 1 | 4 | M | E | 0.385 | 0.181 | 0.016 | 0.803 | 0.445333 |
| 1 | 5 | S | E | 0.479 | 0.268 | 0.043 | 0.689 | 0.516556 |
| 1 | 6 | Q | E | 0.332 | 0.191 | 0.086 | 0.723 | 0.554556 |
| 1 | 7 | L | B | 0.192 | 0.191 | 0.086 | 0.723 | 0.555889 |
| 1 | 8 | E | B | 0.248 | 0.199 | 0.152 | 0.649 | 0.559333 |
| 1 | 9 | R | B | 0.18 | 0.113 | 0.087 | 0.8 | 0.554222 |
| 1 | 10 | N | E | 0.324 | 0.113 | 0.087 | 0.8 | 0.538667 |
| 1 | 11 | I | B | 0.128 | 0.113 | 0.087 | 0.8 | 0.520222 |
| 1 | 12 | E | B | 0.136 | 0.056 | 0.142 | 0.802 | 0.524333 |
| 1 | 13 | T | B | 0.209 | 0.056 | 0.142 | 0.802 | 0.516556 |
| 1 | 14 | I | B | 0.124 | 0.066 | 0.296 | 0.638 | 0.507556 |
| 1 | 15 | I | B | 0.125 | 0.066 | 0.296 | 0.638 | 0.522222 |
| 1 | 16 | N | B | 0.25 | 0.056 | 0.142 | 0.802 | 0.536889 |
| 1 | 17 | T | B | 0.242 | 0.118 | 0.15 | 0.732 | 0.548889 |
| 1 | 18 | F | B | 0.144 | 0.053 | 0.043 | 0.903 | 0.550444 |
| 1 | 19 | H | E | 0.298 | 0.053 | 0.043 | 0.903 | 0.557333 |
| 1 | 20 | Q | B | 0.233 | 0.052 | 0.084 | 0.864 | 0.563 |
| 1 | 21 | Y | B | 0.142 | 0.056 | 0.142 | 0.802 | 0.557333 |
| 1 | 22 | S | B | 0.165 | 0.056 | 0.142 | 0.802 | 0.552444 |
| 1 | 23 | V | B | 0.125 | 0.056 | 0.142 | 0.802 | 0.545444 |
| 1 | 24 | K | B | 0.115 | 0.056 | 0.142 | 0.802 | 0.521111 |
| 1 | 25 | L | B | 0.113 | 0.118 | 0.15 | 0.732 | 0.519333 |
| 1 | 26 | G | B | 0.137 | 0.113 | 0.087 | 0.8 | 0.513778 |
| 1 | 27 | H | E | 0.22 | 0.052 | 0.084 | 0.864 | 0.515889 |
| 1 | 28 | P | B | 0.064 | 0.052 | 0.084 | 0.864 | 0.512333 |
| 1 | 29 | D | B | 0.129 | 0.056 | 0.142 | 0.802 | 0.509556 |
| 1 | 30 | T | B | 0.182 | 0.125 | 0.227 | 0.648 | 0.505333 |
| 1 | 31 | L | B | 0.103 | 0.199 | 0.152 | 0.649 | 0.510667 |
| 1 | 32 | N | B | 0.178 | 0.278 | 0.093 | 0.628 | 0.506778 |
| 1 | 33 | Q | B | 0.118 | 0.386 | 0.097 | 0.517 | 0.507333 |
| 1 | 34 | G | B | 0.082 | 0.386 | 0.097 | 0.517 | 0.492778 |
| 1 | 35 | E | B | 0.093 | 0.386 | 0.097 | 0.517 | 0.470111 |
| 1 | 36 | F | B | 0.081 | 0.386 | 0.097 | 0.517 | 0.457778 |
| 1 | 37 | K | B | 0.063 | 0.561 | 0.047 | 0.393 | 0.444778 |
| 1 | 38 | E | B | 0.083 | 0.561 | 0.047 | 0.393 | 0.440444 |
| 1 | 39 | L | B | 0.061 | 0.66 | 0.049 | 0.291 | 0.433889 |
| 1 | 40 | V | B | 0.108 | 0.751 | 0.05 | 0.199 | 0.414333 |
| 1 | 41 | R | B | 0.062 | 0.717 | 0.014 | 0.269 | 0.415667 |
| 1 | 42 | K | B | 0.096 | 0.622 | 0.015 | 0.363 | 0.414333 |
| 1 | 43 | D | B | 0.118 | 0.522 | 0.016 | 0.462 | 0.405 |
| 1 | 44 | L | B | 0.081 | 0.522 | 0.016 | 0.462 | 0.402 |
| 1 | 45 | Q | B | 0.118 | 0.522 | 0.016 | 0.462 | 0.409111 |
| 1 | 46 | N | B | 0.198 | 0.522 | 0.016 | 0.462 | 0.425667 |
| 1 | 47 | F | B | 0.087 | 0.522 | 0.016 | 0.462 | 0.429778 |
| 1 | 48 | L | B | 0.1 | 0.622 | 0.015 | 0.363 | 0.442889 |
| 1 | 49 | K | B | 0.162 | 0.622 | 0.015 | 0.363 | 0.464556 |
| 1 | 50 | K | B | 0.146 | 0.622 | 0.015 | 0.363 | 0.467111 |
| 1 | 51 | E | B | 0.138 | 0.522 | 0.016 | 0.462 | 0.480778 |
| 1 | 52 | N | E | 0.321 | 0.455 | 0.046 | 0.498 | 0.502556 |
| 1 | 53 | K | B | 0.196 | 0.354 | 0.048 | 0.598 | 0.518111 |
| 1 | 54 | N | E | 0.269 | 0.184 | 0.043 | 0.773 | 0.514222 |
| 1 | 55 | E | B | 0.127 | 0.191 | 0.086 | 0.723 | 0.520556 |
| 1 | 56 | K | B | 0.193 | 0.113 | 0.087 | 0.8 | 0.528 |
| 1 | 57 | V | B | 0.161 | 0.118 | 0.15 | 0.732 | 0.527222 |
| 1 | 58 | I | B | 0.141 | 0.118 | 0.15 | 0.732 | 0.525556 |
| 1 | 59 | E | B | 0.201 | 0.052 | 0.084 | 0.864 | 0.532889 |
| 1 | 60 | H | E | 0.343 | 0.018 | 0.088 | 0.893 | 0.527111 |
| 1 | 61 | I | B | 0.119 | 0.064 | 0.216 | 0.721 | 0.515444 |
| 1 | 62 | M | B | 0.19 | 0.064 | 0.216 | 0.721 | 0.519444 |
| 1 | 63 | E | B | 0.132 | 0.064 | 0.216 | 0.721 | 0.527333 |
| 1 | 64 | D | B | 0.122 | 0.064 | 0.216 | 0.721 | 0.531222 |
| 1 | 65 | L | B | 0.088 | 0.064 | 0.216 | 0.721 | 0.518444 |
| 1 | 66 | D | B | 0.109 | 0.056 | 0.142 | 0.802 | 0.520333 |
| 1 | 67 | T | B | 0.187 | 0.113 | 0.087 | 0.8 | 0.529111 |
| 1 | 68 | N | E | 0.295 | 0.113 | 0.043 | 0.844 | 0.532333 |
| 1 | 69 | A | B | 0.171 | 0.191 | 0.086 | 0.723 | 0.533889 |
| 1 | 70 | D | B | 0.13 | 0.113 | 0.087 | 0.8 | 0.538222 |
| 1 | 71 | K | B | 0.161 | 0.113 | 0.087 | 0.8 | 0.528889 |
| 1 | 72 | Q | B | 0.228 | 0.113 | 0.087 | 0.8 | 0.532111 |
| 1 | 73 | L | B | 0.105 | 0.113 | 0.087 | 0.8 | 0.521333 |
| 1 | 74 | S | B | 0.257 | 0.113 | 0.087 | 0.8 | 0.527222 |
| 1 | 75 | F | B | 0.149 | 0.184 | 0.043 | 0.773 | 0.522778 |
| 1 | 76 | E | B | 0.149 | 0.278 | 0.093 | 0.628 | 0.511111 |
| 1 | 77 | E | B | 0.153 | 0.184 | 0.043 | 0.773 | 0.502778 |
| 1 | 78 | F | B | 0.118 | 0.278 | 0.093 | 0.628 | 0.494778 |
| 1 | 79 | I | B | 0.124 | 0.307 | 0.165 | 0.527 | 0.496 |
| 1 | 80 | M | B | 0.22 | 0.278 | 0.093 | 0.628 | 0.503556 |
| 1 | 81 | L | B | 0.131 | 0.278 | 0.093 | 0.628 | 0.489778 |
| 1 | 82 | M | B | 0.223 | 0.278 | 0.093 | 0.628 | 0.486556 |
| 1 | 83 | A | B | 0.182 | 0.199 | 0.152 | 0.649 | 0.480889 |
| 1 | 84 | R | B | 0.102 | 0.113 | 0.087 | 0.8 | 0.477889 |
| 1 | 85 | L | B | 0.111 | 0.113 | 0.087 | 0.8 | 0.477889 |
| 1 | 86 | T | B | 0.237 | 0.113 | 0.087 | 0.8 | 0.485556 |
| 1 | 87 | W | B | 0.16 | 0.184 | 0.043 | 0.773 | 0.500667 |
| 1 | 88 | A | B | 0.229 | 0.339 | 0.016 | 0.645 | 0.520333 |
| 1 | 89 | S | E | 0.435 | 0.339 | 0.016 | 0.645 | 0.532889 |
| 1 | 90 | H | E | 0.343 | 0.257 | 0.016 | 0.727 | 0.545444 |
| 1 | 91 | E | B | 0.183 | 0.184 | 0.043 | 0.773 | 0.550222 |
| 1 | 92 | K | B | 0.203 | 0.118 | 0.15 | 0.732 | 0.551333 |
| 1 | 93 | M | E | 0.301 | 0.064 | 0.216 | 0.721 | 0.556222 |
| 1 | 94 | H | E | 0.294 | 0.02 | 0.205 | 0.775 | 0.557778 |
| 1 | 95 | E | B | 0.093 | 0.02 | 0.205 | 0.775 | 0.542222 |
| 1 | 96 | G | B | 0.079 | 0.02 | 0.205 | 0.775 | 0.523333 |
| 1 | 97 | D | B | 0.072 | 0.021 | 0.279 | 0.699 | 0.496778 |
| 1 | 98 | E | B | 0.064 | 0.021 | 0.279 | 0.699 | 0.490889 |
| 1 | 99 | G | B | 0.055 | 0.022 | 0.359 | 0.619 | 0.493556 |
| 1 | 100 | P | B | 0.062 | 0.021 | 0.451 | 0.528 | 0.495889 |
| 1 | 101 | G | B | 0.125 | 0.021 | 0.279 | 0.699 | 0.502556 |
| 1 | 102 | H | E | 0.249 | 0.019 | 0.141 | 0.84 | 0.503222 |
| 1 | 103 | H | B | 0.255 | 0.019 | 0.141 | 0.84 | 0.503222 |
| 1 | 104 | H | B | 0.198 | 0.021 | 0.279 | 0.699 | 0.505333 |
| 1 | 105 | K | B | 0.151 | 0.021 | 0.451 | 0.528 | 0.512111 |
| 1 | 106 | P | B | 0.088 | 0.021 | 0.451 | 0.528 | 0.531889 |
| 1 | 107 | G | B | 0.124 | 0.021 | 0.451 | 0.528 | 0.529778 |
| 1 | 108 | L | B | 0.173 | 0.021 | 0.451 | 0.528 | 0.531556 |
| 1 | 109 | G | B | 0.206 | 0.022 | 0.359 | 0.619 | 0.525556 |
| 1 | 110 | E | E | 0.298 | 0.021 | 0.279 | 0.699 | 0.465667 |
| 1 | 111 | G | B | 0.268 | 0.02 | 0.205 | 0.775 | 0.407 |
| 1 | 112 | T | E | 0.506 | 0.019 | 0.141 | 0.84 | 0.349333 |
| 1 | 113 | P | E | 0.734 | 0.003 | 0.003 | 0.994 | 0.297667 |

**Supplementary table 4:** The epitopes predicted by ABCred server for HMGB1, S100A8 and S100A9 protein.

| **Protein** | **Rank** | **Sequence** | **Start position** | **Score** |
| --- | --- | --- | --- | --- |
| HMGB-1 | 1 | CREEHKKKHPDASVNF | 30 | 0.94 |
|  | 2 | GSSGMGKGDPKKPRGK | 4 | 0.92 |
|  | 2 | AFFVQTCREEHKKKHP | 24 | 0.92 |
|  | 3 | YEREMKTYIPPKGETK | 78 | 0.87 |
|  | 4 | PPKGETKKKFKDPNAP | 87 | 0.84 |
|  | 5 | PDASVNFSEFSKKCSE | 39 | 0.8 |
|  | 6 | SERWKTMSAKEKGKFE | 53 | 0.79 |
|  | 6 | NTAADDKQPYEKKAAK | 142 | 0.79 |
|  | 7 | SAKEKGKFEDMAKADK | 60 | 0.74 |
|  | 8 | GEHPGLSIGDVAKKLG | 122 | 0.73 |
|  | 9 | CSEYRPKIKGEHPGLS | 113 | 0.71 |
|  | 9 | KGDPKKPRGKMSSYAF | 10 | 0.71 |
|  | 10 | PRGKMSSYAFFVQTCR | 16 | 0.7 |
|  | 11 | NAPKRPPSAFFLFCSE | 100 | 0.68 |
|  | 12 | YEKKAAKLKEKYEKDI | 151 | 0.65 |
|  | 13 | LKEKYEKDIAAYRAKG | 158 | 0.56 |
| S100A8 | 1 | TECPQYIRKKGADVWF | 40 | 0.93 |
|  | 2 | YSLIKGNFHAVYRDDL | 19 | 0.77 |
|  | 3 | NSIIDVYHKYSLIKGN | 10 | 0.7 |
|  | 4 | IRKKGADVWFKELDIN | 46 | 0.68 |
|  | 5 | VWFKELDINTDGAVNF | 53 | 0.62 |
| S100A9 | 1 | EKMHEGDEGPGHHHKP | 91 | 0.89 |
|  | 1 | IEHIMEDLDTNADKQL | 58 | 0.89 |
|  | 2 | EGPGHHHKPGLGEGTP | 98 | 0.84 |
|  | 3 | ARLTWASHEKMHEGDE | 83 | 0.76 |
|  | 4 | TIINTFHQYSVKLGHP | 13 | 0.73 |
|  | 5 | SVKLGHPDTLNQGEFK | 22 | 0.69 |
|  | 6 | QLSFEEFIMLMARLTW | 72 | 0.59 |


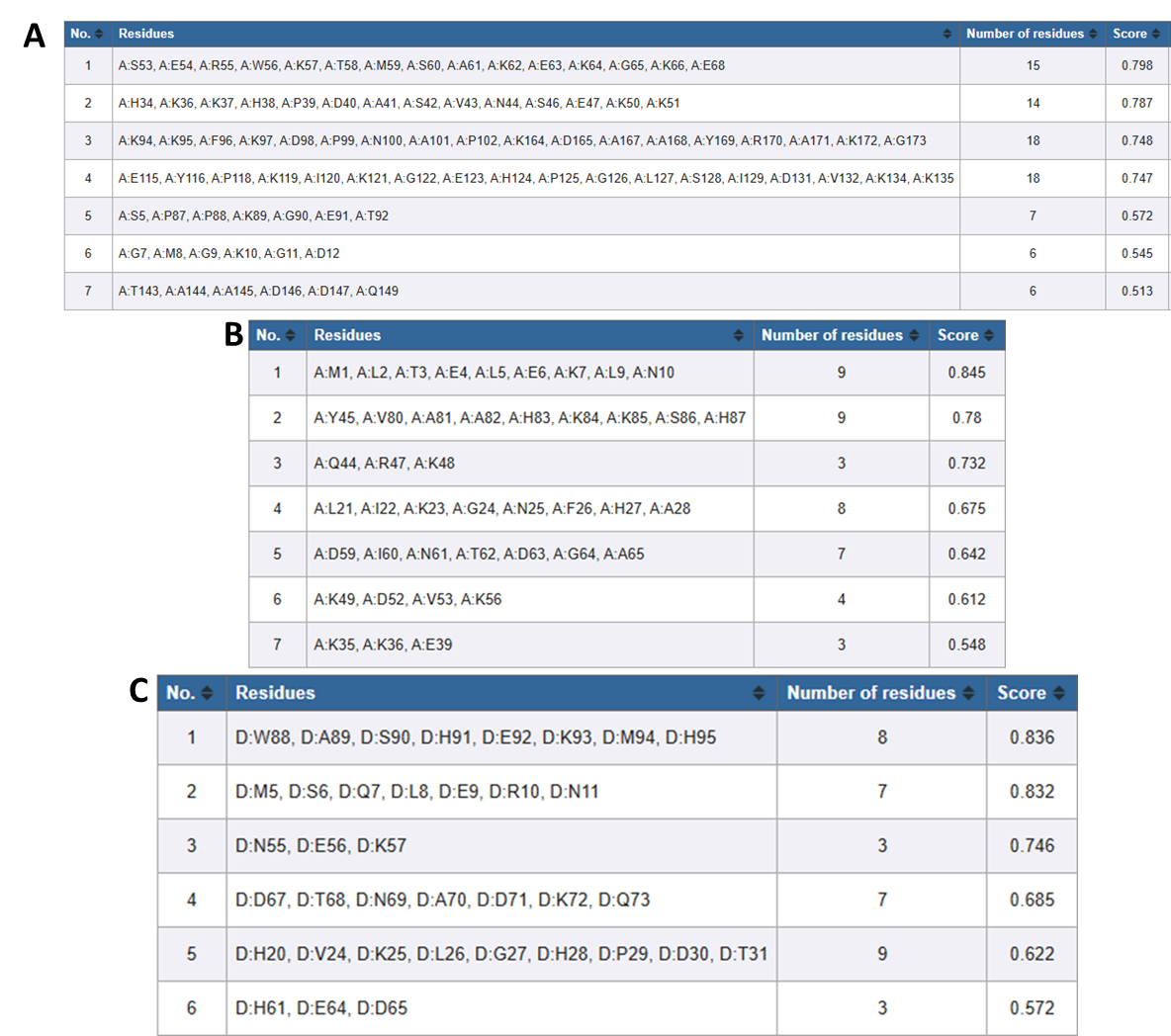


**Supplementary Figure 2:** Conformational epitopes predicted by Ellipro server. A: HMGB-1; B: S100A8 and C: S100A9
